# Occurrence and health risk assessment of PFAS and possible precursors: a case study in a drinking water treatment plant and bottled water (south Catalonia, Spain)

**DOI:** 10.1007/s11356-024-34805-6

**Published:** 2024-09-13

**Authors:** Joana Martínez, Massimo Picardo, Alejandra Peñalver, Josepa Fabregas, Carme Aguilar, Francesc Borrull

**Affiliations:** 1 Departament de Química Analítica i Química Orgànica, Universitat Rovira i Virgili, Unitat de Radioquímica Ambiental i Sanitaria, Ctra. Nacional 340, Km. 1094, 43895 L’Ampolla, Tarragona, Spain; 2 Consorci d’Aigües de Tarragona, Ctra. Nacional 340, Km. 1094, 43895 L’Ampolla, Tarragona, Spain; 3 Serra Húnter Professor, Generalitat de Catalunya, Barcelona, Spain

**Keywords:** PFAS, PFAS precursor, Drinking water, TOP assay, Exposure assessment, DWTP

## Abstract

The presence of PFAS in drinking water may pose a serious threat to human health. This study aims to determine the levels of these compounds and their precursors in water samples from a drinking water treatment plant (DWTP) located in l’Ampolla (Spain) and to assess their fate. Additionally, ten Spanish bottled waters were analyzed to compare the occurrence of PFAS in the mentioned matrices and in drinking water. Off-line solid phase extraction (SPE) followed by liquid chromatography-tandem mass spectrometry (HPLC–MS/MS) was applied to determine 26 PFAS and PFAS precursors after a total oxidizable precursor assay. The analytical method presents low quantification limits (0.25–5 ng/L). A total PFAS concentration of up to 16 ng/L in all the DWTP samples was obtained, and 6:2 FTS was the only precursor detected. Results are close to the quantification limits, resulting in a high degree of uncertainty, and for this, it is difficult to evaluate the DWTP PFAS removal efficiency. Regarding bottled water, total PFAS concentration found was up to 12 ng/L in one of ten samples, with no precursors detected. Exposure assessment revealed that there is no risk associated with the ingestion of the samples analyzed. Moreover, there were no differences in terms of risk between drinking water from l’Ampolla DWTP and bottled water.

**Graphical abstract:**

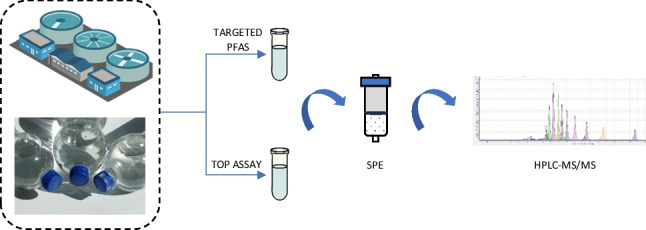

## Introduction

Per- and polyfluoroalkyl substances (PFAS), known as “forever chemicals” are a wide group of more than 7 million chemicals, including salts and mixtures. These compounds consist of a hydrophobic alkyl chain of variable length depending on the number of carbons (European Environment Agency 2022; Schymanski et al. 2023). This carbon chain could be totally or partially fluorinated, ending with a hydrophilic group. Their properties make PFAS useful in a wide range of processes and applications such as paints, textiles, and food packaging (Glüge et al. 2020; The Interestate Technology and Regulatory Council 2023). Due to their large-scale production and their widespread use, PFAS have reached the environment and tend to bioaccumulate in wildlife and human beings (Lasier et al. 2011; Lee et al. 2020; Poothong et al. 2020). Nowadays, several studies reveal negative effects on reproductive toxicity, neurotoxicity, immunotoxicity, and carcinogenicity, among others (Pizzurro et al. 2019; Pilli et al. 2021), which provide fundamentals for regulatory authorities to regulate PFAS compounds. Driven by different reports on toxic effects, in 2008, the European Food Safety Authority (EFSA) established, and recently revised, a tolerable weekly intake (TWI) for the sum of perfluorooctanoic acid (PFOA), perfluorooctanesulfonic acid (PFOS), perfluorononanoic acid (PFNA), and perfluorohexanesulfonic acid (PFHxS) with a value of 4.4 ng/kg_bw_ per week (Schrenk et al. 2020).

Drinking water is one of the routes of human exposure to PFAS through dietary intake, other being dust, indoor air inhalation, and dermal contact (Poothong et al. 2020). Because of that, PFAS is a topic of concern in the scientific community, and several research studies related to occurrence, fate in environment, and in drinking and wastewater treatment plants, as well as on human health exposure to these compounds in surface and drinking water, have been published to date. The occurrence of PFAS in surface water could be an indicator of their potential presence in drinking water (McMahon et al. 2022) since some studies in the literature suggested that, in general, drinking water treatment plants (DWTP) applying conventional treatment steps like coagulation, flocculation, sedimentation, and filtration do not tend to effectively remove PFAS, even though other studies have demonstrated that advanced treatment processes like activated carbon, ion exchange resins, nanofiltration, and reverse osmosis may be effective in removing these compounds (Rahman et al. 2014; Sun et al. 2016; Kim et al. 2020).

Although PFAS generally occur at low concentrations in environmental waters, their occurrence and risk is a growing worldwide concern, and in this sense, these compounds have been regulated in the European Drinking Water Directive, with a limit of 500 ng/L for all PFAS (Organization for Economic Cooperation and Development (OECD) 2023). This Directive has been transposed into Spanish legislation, in particular in Royal Decree 3/2023, where the parametrical value for all PFAS is 100 ng/L; a parametrical value of 70 ng/L for perfluorobutanoic acid (PFBA), PFHxS, PFOA and PFNA, individually, has been also established (Ministerio de la Presidencia R con las C y MD 2023).

The increasing concern regarding PFAS substances in water samples implies the need for the development of analytical methods and, consequently, different approaches to determine PFAS in water have been reported in recent years by several authors (Schultz et al. 2006; Ciofi et al. 2018; Borrull et al. 2020; Mottaleb et al. 2021). All of these are generally based on liquid chromatography (HPLC) coupled with triple quadrupole (QqQ), time-of-flight (QTOF), and Quadrupole-Orbitrap mass spectrometry, as Teymoorian et al (2022) presented in their recent research review work.

There are different strategies currently in use for sample pretreatment. While some authors use direct injection or large volume injection of water samples (between 0.1 and 0.9 mL Ciofi et al. 2018; Borrull et al. 2020; Mottaleb et al. 2021)), the most common strategy is to include an SPE procedure (between 100 and 5000 mL (Teymoorian et al. 2022)). Direct injection of water samples is used in some cases but, even though this is a quick way to determine PFAS (Schultz et al. 2006; Ciofi et al. 2018; Borrull et al. 2020; Mottaleb et al. 2021), solid phase extraction (SPE) is the most used strategy to be able to reach the usual PFAS levels in environmental water samples (sub-ng/L levels). Regarding SPE cartridge types, the most used is anion exchange mix-mode sorbent > weak anion exchange sorbent > lipophilic-hydrophilic balance sorbent (HLB) (Teymoorian et al. 2022), and the reported recoveries are in the range of 70–110% for most of the carboxylic and sulfonate PFAS (Kaboré et al. 2018; Coggan et al. 2019; Chen et al. 2021; Jurikova et al. 2022).

The scientific community is also working on determining PFAS precursors, also referred to as “hidden PFAS.” This is a challenging task for various reasons, as the precursors are unknown, they may degrade, and there are no available standards (Al Amin et al. 2020). Given this, total oxidizable precursor (TOP) assay is applied—with persulfate and basic conditions—and it is based on the oxidation of PFAS precursors into perfluoroalkyl acids (PFAAS), which can be easily determined by the HPLC–MS/MS target method. Knowing the PFAS concentration before and after TOP, it is then possible to assess the total concentration of PFAS precursors in samples (Houtz and Sedlak 2012; Martin et al. 2019; Nolan et al. 2019; Kaiser et al. 2021; Nikiforov 2021). This assay has been applied to different environmental matrices such as soil, biota, or water. For example, Martin et al. (2019) reveals the presence of PFAS precursors such as 6:2 fluorotelomer sulfonamide alkylbetaine (6:2 FTAB), 6:2 fluorotelomer sulfonate (6:2FTS), 8:2 fluorotelomer sulfonate (8:2FTS), perfluorooctanesulfonamide (PFOSAm), and perfluorohexanesulfonamide (FHxSA) in groundwater samples from fire-equipment testing sites in Canada after performing TOP.

The above-mentioned analytical methodologies have been widely applied to monitor PFAS presence in drinking waters. In this sense, elevated concentrations of PFAS (between 20 and 20,000 ng/L) have been reported, particularly near industrial or fire-training activities sites (Mussabek et al. 2023). On the contrary, PFAS in drinking water worldwide ranges from 0.08 to 11.27 ng/L (Turkey) and to 300 ng/L (Czech Republic) in terms of total concentration and from 0.48 ng/L (Turkey) to 18.9 ng/L (USA) in bottled water (Teymoorian et al. 2022).

Given the context presented above, the aim of the present study is to apply a method based on off-line SPE HPLC–MS/MS to determine 26 PFAS at ng/L levels in selected DWTP water samples from different treatment processes as well as surface water (Ebro River water) and drinking water. DWTP water samples were collected to assess PFAS fate and to analyze the removal effectiveness of a DWTP equipped with advanced treatment processes. Moreover, another aim was to have an overview of the PFAS contamination status in bottled water, as it is an important source of drinking water in Spain, and to compare the occurrence of these compounds in the mentioned matrix and in drinking water. Total oxidizable assay (TOP) was performed to have an estimation of the number of possible PFAS precursors (non-target compounds in the analytical method) present in samples as they can contribute to increasing the total final PFAS concentration. Finally, an assessment has been performed of exposure and human health risk from targeted PFAS due to ingestion of l’Ampolla DWTP drinking water and bottled water.

## Materials and methods

### Reagents and standards

High purity chemical standards of target PFAS were purchased from AccuStandard (New Haven, USA) (a mix standard of 24 PFAS) and Wellington Laboratories (Whitby, ON, Canada) (two individual PFAS). Moreover, a mix standard of 13 isotopically labelled PFAS was obtained from Wellington Labs, Inc. (Whitby, ON, Canada) and was used as surrogate standards. The 26 target and 13 isotopically labelled PFAS acronyms, retention time, and MRM mass transitions are shown in Table 1.

**Table 1:** Acronyms, retention time, and MRM mass transitions of target and isotopically labelled PFAS standards (ISTD) of the target analytes investigated in this study

Analytes	*t*_R_ (min)	Precursor ion (m/z)	Quantifier		Qualifier	
			(m/z)	Collision energy 1 (V)	(m/z)	Collision energy 2 (V)
PFBA	6.9	213	169	4	-	-
^13^C PFBA	7.0	217	172	4		
PFPeA	7.8	263	219	4	-	-
^13^C PFPA	7.8	268	223	4		
4:2FTS^1^	7.8	327	306.9	24	80.9	44
PFHxA	8.6	313	269	4	119	18
^13^C PFHxA	8.6	318	273	4		
6:2FTS^1^	9.0	427	407	24	81	30
^13^C PFBS	9.1	302	80	40		
PFBS	9.2	299	99	30	80	40
PFHpA	9.2	363	319	6	169	18
^13^C PFHpA	9.3	367	322	6		
PFPS^2^	9.7	349	99	35	80	39
PFOA	9.9	413	369	6	169	16
^13^C PFOA	10.0	421	376	6		
PFOSA^1^	10.1	497.9	77.9	40	47.9	96
8:2FTS^1^	10.2	527	506.8	28	-	-
PFHxS	10.3	399	119	48	99	42
^13^C PFHxS	10.3	402	80	42		
PFNA	10.5	463	419	6	219	14
^13^C PFNA	10.5	472	427	6		
PFHpS^2^	10.8	449	169	46	80	50
PFDA	11.1	513	469	8	219	16
^13^C PFDA	11.1	519	474	8		
PFOS	11.3	499	99	46	80	50
^13^C PFOS	11.3	507	80	50		
N-MetFOSAA^1^	11.4	570	482.9	12	419	20
PFNS^1^	11.6	549	99	56	80	53
PFUnDA	11.7	563	519	6	319	15
^13^C PFUnDA	11.7	570	525	6		
PFDS^1^	12.0	599	99	57	80	54
N-EtFOSAA^1^	12.0	584	483	16	418.9	20
PFDoA	12.2	613	569	8	169	26
^13^C PFDoA	12.2	615	570	8		
PFUnS^1^	12.3	649	99	80	80	120
PFDoS^1^	12.6	699	99	40	80	100
PFTrDA^3^	12.7	663	619	9	169	86
PFTrS^4^	13.0	749	99	95	80	200
^13^C PFTeDA	13.1	715	670	10		

The compounds with superindex do not have analogue ISTD: 1: ^13^C PFOS; 2: ^13^C PFHxS; 3: ^13^CPFDoA; 4: ^13^C PFTeDA

A working standard solution containing 26 target PFAS was prepared with methanol (MeOH) to a final concentration of 5000 ng/L and then stored at − 20 °C in amber glass vials. The same procedure was applied to get a working standard solution of isotopically labelled PFAS to a final concentration of 5000 ng/L.

HPLC water, MeOH, acetonitrile (ACN), ammonium acetate, and formic acid were of HPLC grade and were obtained from Honeywell (Seelze, Germany). Potassium persulfate (> 99%) was acquired from Sigma-Aldrich (St. Louis, MO, USA). Sodium hydroxide (NaOH) was acquired from J.T. Baker (Gliwice, Poland), and hydrochloric acid (HCl) was obtained from Supelco (Darmstadt, Germany).

Bond Elut PFAS WAX SPE cartridges (150 mg/6 mL, 50 µm) purchased from Agilent Technologies (USA) were used for the extraction procedure, as well as regenerated cellulose syringe filters (13 mm Ø, 0.45 µm) from LLG Labware (USA).

### Sample collection and preparation

**Fig. 1:**
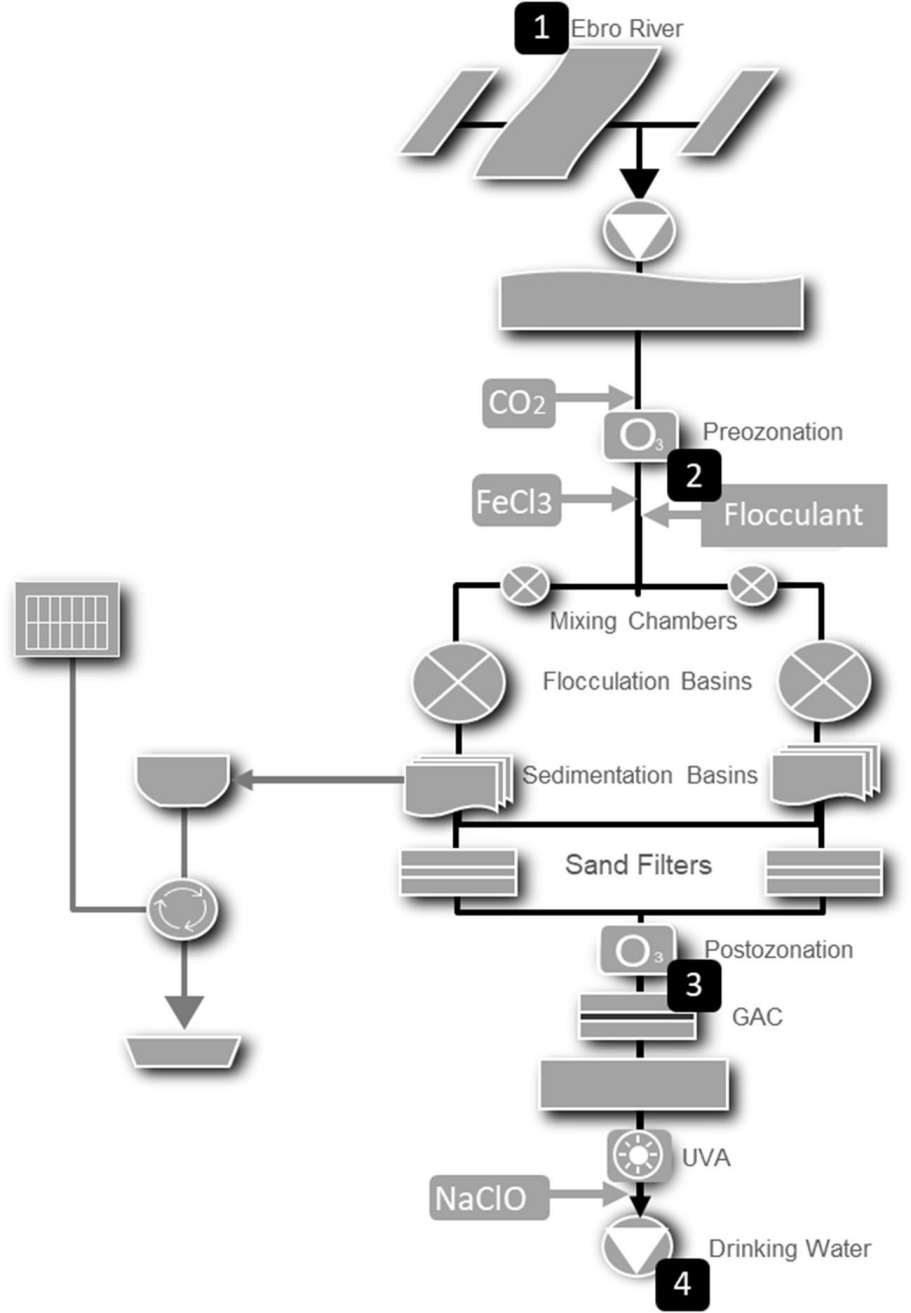
Schematic of the DWTP processes. 1 to 4 are the selected sampling points

Water samples were collected, each month, from l’Ampolla DWTP between January and November 2022. This DWTP, with the capacity to treat up to 4 m^3^/s of water, comprises the following treatment steps: CO_2_ pH adjustment, pre-ozonation, coagulation-flocculation-decantation, sand filtration, post-ozonation, granular-activated carbon filtration (GAC), ultra-violet disinfection (UV), and NaClO chlorination. Figure 1, which is a schematic of l’Ampolla DWTP processes, shows the sampling points established in the present study: (1) at DWTP influent (water from Ebro River), (2) after pre-ozonation, (3) after post-ozonation, and (4) at DWTP drinking water (after final chlorination with NaClO).

Water samples were collected in 500 mL high density polyethylene (HDPE) plastic bottles. After collection, all samples were stored at 3 ± 2 °C until analysis.

Ten bottled water samples from different commercial brands were purchased from local supermarkets of l’Ampolla, Tarragona. After that, samples were also stored at 3 ± 2 °C until analysis.

An intercomparison sample with 32 native compounds was obtained from Round 306 of ERA’s Water Supply for Drinking Water PT Scheme.

#### SPE method

Before assay, each sample was divided in two. One half was used for determining 26 target PFAS by SPE method and the other half to perform TOP assay. All samples were analyzed in triplicate.

The extraction method was based on off-line SPE. One hundred milliliters of sample, after the addition of the mixture of isotopically labelled surrogates in methanol and its acidification with 1.25 mL of formic acid (pH < 3), was loaded onto the Agilent Bond Elut PFAS WAX cartridge. The cartridges were pre-conditioned with 5 mL of 3% NH_4_OH in 60/40 ACN/MeOH, 5 mL of HPLC water with 0.3 M of formic acid. Then, samples were loaded into cartridges and rinsed with 5 mL HPLC water. Finally, target analytes were eluted into Falcon tubes with 5 mL of 3% NH_4_OH in 60/40 ACN/MeOH. The extract was evaporated using a vacuum centrifugal concentrator, IR Micro-Cenvac (N-Biontek, Korea) to a final volume of 500 µL (Hunt et al. 2018). Finally, it was adjusted to 1 mL with ACN:H_2_O (1:1) and filtered using a 0.22-µm cellulose regenerated syringe filter before injection.

#### TOP-SPE method

TOP assay was conducted according to Houtz and Sedlak (2012) and Martin et al. (2019), with some modifications. Briefly, a 100 mL water sample aliquot was placed in a 100 mL HDPE bottle and a final concentration 0.18 M of potassium persulfate and 0.45 M of NaOH was added. Samples were heated in a water bath for 6 h at 85 °C, shaking it regularly. The pH of each sample was measured before and after sample heating to confirm it was maintained during TOP assay (pH > 12). After that, samples were cooled down to ambient temperature in an ice bath, and pH was brought to approximately 8 with HCl to stop the reaction. Then, the final extract was acidified with formic acid (pH < 3) and subjected to SPE as has been explained in the “SPE method” section.

### Equipment and chromatographic conditions

The analysis of the obtained extracts was performed using an Agilent chromatographic system (Agilent Technologies, Waldbronn, Germany) equipped with a 1260 HiP degassing unit, 1260 binary pump, 1260 multisampler, and a 1260 thermostated column compartment. The target PFAS were chromatographically separated with a Poroshell 120 EC-C18 analytical column (50 mm × 3 mm, 2.7 µm) and a 120 EC-C18 guard cartridge (5 mm × 3 mm × 2.7 µm). Water with 10 mM ammonium acetate (A) and ACN (B) was used as mobile phase. The gradient profile started at 10% B, which was raised to 40% B within 2 min, then to 95% B in 4 min and then 100% B in 6.5 min. Finally, it returned to initial conditions (10% B) in 0.5 min and held for 5 min to equilibrate the column for the next analysis. A flow rate of 0.40 mL/min was used and the temperature of the chromatographic column was kept at 45 °C. The injection volume was 100 µL. The acquisition was performed in dynamic multiple reaction monitoring (dMRM) in negative mode using an ESI source. The optimal conditions for the analytes were as follows: a nitrogen flow rate of 8 mL/min, a nebulizer pressure of 30 psi (N_2_) and source temperature of 300 °C. The collision energies for all compounds were from 4 to 200 V. The optimal values of HPLC–MS/MS parameters are summarized in Table 1.

To reduce background contamination, an analytical LC column (Poroshell 120 EC-C18, 100 mm × 4.6 mm × 2.7 µm) was installed between the mobile phase mixing chamber and the sample injector as a delay column. Moreover, all polyeterketone tubes (PEEK) were substituted by stainless steel ones.

### Method performance evaluation

The validation of this method followed the EPA validation guideline for PFAS method (United States Environmental Protection Agency 2018), covering several aspects including linearity, detection and quantification limits (LOD and LOQ), repeatability, accuracy, matrix effect, recovery, and trueness which was evaluated through *z*-scores. Analogue isotopically labelled standards (ISTD) were used for almost all target compounds. However, for some compounds where the analogue ISTD was unavailable, surrogate-corrected recovery of the extraction procedure and surrogate-corrected matrix effect were assessed.

The SPE extraction step recovery (% RE) was calculated as the ratio of the peak areas for a sample spiked at a concentration of 100 ng/L of target analytes and 50 ng/L of ISTD before SPE extraction compared to the ratio of the peak areas obtained from the sample spiked at the same concentrations after SPE.

For some PFAS compounds, no isotopically labelled standards analogues were available. For this reason, their quantification has been performed by the interpolation of the signal in the calibration curve (without using ISTD).

The surrogate-corrected matrix effect (% ME) was also assessed. It was calculated as the ratio between the analyte signal response obtained from the spiked sample matrix compared with that obtained when a neat sample is spiked. Matrix effect factor is considered acceptable when it is between the range of 20 and − 20%.

Limits of detection (LODs) correspond to the concentration whose signal-to-noise (S/N) ratio is greater than 3, and limits of quantification (LOQs) were defined as the lowest point of the calibration curve with S/N ≥ 10.

The repeatability of the method was expressed as relative standard deviation (% RSD) of five replicates at a concentration of 5 ng/L, 50 ng/L, and 100 ng/L.

TOP assay has been performed to assess the presence of PFAS precursors in water samples. It is necessary to evaluate the oxidation efficiency of the applied assay by spiking surface water from the Ebro River with 6:2 FTS (which is a PFAS precursor). Briefly, 10 µL of 1 µg/mL of 6:2FTS standard was added to a 100 mL polyethylene bottle and evaporated to dryness without heating. After that, it was redissolved with 100 mL of surface water, and it was subjected to the TOP-SPE method (“TOP-SPE method” section).

### Exposure and human health risk assessment

The presence of PFAS in drinking water turns water ingestion into a potential exposure route for these compounds to reach our organism. Thus, in 2020, the EFSAs CONTAM Panel (Panel on Contaminants in the Food Chain), decided to consider the sum of PFOA, PFNA, PFHxS, and PFOS as main contributors when estimating the possibility of these compounds causing adverse health effects by calculating the estimated weekly intake (EWI).

1 $$\text{EWI}= \frac{\sum {C}_{\text{PFOA},\text{ PFNA},\text{ PFHxS},\text{ PFOS} }*\text{ DWI}*\text{ FOC}}{\text{BW}}$$

where ∑*C*_PFOA, PFNA, PFHxS, PFOS_ represents the sum of the average measured concentrations in ng/L (considering LOQs for non-detected compounds), DWI (L/day) is the drinking water intake (2 L/day for adults, 1 L/day for children and 0.75 L/day for bottled-fed infants), FOC is the frequency of water consumption (7 day/week), and BW (kg) represents body weight (60 kg for adults, 10 kg for children, and 5 kg for bottled-fed infants). The DWI and BW data were obtained from the WHO Guidelines for drinking water quality (World Health Organization 2022).

For the health risk assessment, hazard quotients (HQ) for the aforementioned four PFAS were calculated according to Eq. (2) (Dvorakova et al. 2023):

2 $$HQ=\frac{EWI}{RfD}$$

where RfD is the reference dose, considered as TWI for ∑4 PFAS = 4.4 ng/kg_bw_/week and is related to the oral exposure to the human population without appreciable risk of negative effects during lifetime exposure.

The interpretation of the HQ value obtained is as follows: HQ < 0.1, indicates no hazard; HQ = 0.1–1.0, indicates a low hazard; HQ > 1–10, indicates a moderate hazard; HQ > 10 indicates a high hazard.

## Results and discussion

### Method validation

The presented analytical method was successfully validated with the parameters described in the “Method performance evaluation” section by SPE and HPLC–MS/MS using drinking water and Ebro River water. The extraction procedure, surrogate-corrected recovery (% RE), surrogate-corrected matrix effect (% ME), as well as linearity, LODs, and LOQs, and the repeatability were evaluated.

The surrogate-corrected % RE and % ME were evaluated for 100 mL of drinking water and Ebro River water using samples spiked with targeted PFAS at 100 ng/L and ISTD at 50 ng/L. However, for some target compounds, there was no availability of the analogue ISTD. The results for these two parameters are shown in Table 2. The % RE of almost all analytes in both matrices are similar and fall within the 70 to 130% range required by EPA methods (United States Environmental Protection Agency 2018). In the case of N-EtFOSAA, PFUnS, and PFTrS, surrogate-corrected recoveries in both matrices were not considered acceptable despite the use of non-analogue ISTD. In these specific situations, their quantification has been performed by the interpolation of the signal in the calibration curve. The results obtained were satisfactory and useful for studying possible analyte losses or contamination during sample preparation. Surrogate-corrected matrix effect was calculated as the ratio of the signal of each analyte spiked in the Ebro River water and drinking water after the SPE extraction and their signal for standard sample solutions. As shown in Table 2, most compounds have |% ME|< 20%, indicating that the matrix effect is not significant and that good recoveries were achieved from both water sample matrices. However, for 4:2 FTS, 8:2 FTS, N-MetFOSAA, and N-EtFOSAA surrogate-corrected |% ME| exceeded 20%, possibly due to the use of non-analogue ISTD. In these situations, % ME are not considered acceptable, and as explained before, their quantification has been performed by the interpolation of the signal in the calibration curve.

**Table 2:** Surrogate-corrected recovery (% RE) and surrogate-corrected matrix effect (% ME) at 100 ng/L of target PFAS in river water and drinking water with SPE and HPLC–MS/MS

Analytes	Ebro River water		Drinking water	
	RE %	ME %	RE %	ME%
PFBA	71	25	95	22
PFBS	130	3	74	9
PFDA	90	8	105	13
PFDoA	91	1	104	2
PFDoS	92	− 6	100	− 6
PFDS	114	− 9	103	− 20
PFHpA	87	27	116	27
PFHpS	90	− 4	111	− 9
PFHxA	98	4	102	7
PFHxS	100	14	110	4
PFNA	99	− 5	100	− 5
PFNS	117	− 20	81	− 18
PFOA	67	20	74	20
PFOS	103	4	97	0
PFOSA	68	26	130	24
PFPeA	103	18	116	22
PFPS	113	− 17	85	− 13
PFTrA	73	27	113	25
PFTrS	124	− 35	43	− 27
PFUnDA	97	9	104	9
PFUnS	130	10	44	22
4:2 FTS	85	− 30	74	− 51
6:2 FTS	134	− 12	131	− 24
8:2 FTS	124	− 39	126	− 47
N-EtFOSAA	48	171	238	219
N-MetFOSAA	76	81	132	87

RSD % (*n* = 5) for RE% and ME% in Ebro River water and drinking water is < 15%

Curves were plotted, and the linear ranges of calibration for each target PFAS compound are shown in Table 3. Linearity was good for all the compounds (*R*^2^ > 0.996), except for 6:2 FTS, N-MetFOSAA, N-EtFOSAA, PFDS, and PFTrS, which coefficients ranged between 0.93 and 0.98 due to the dispersion of the obtained results. The LODs were between 0.1 and 1.7 ng/L and LOQs (the lowest point of each calibration curve) were between 0.25 and 5 ng/L for both matrices because the % RE and % ME were similar in both cases. Taking into account current guidelines (Ministerio de la Presidencia R con las C y MD 2023), the obtained limits were satisfactory to assess the presence of the mentioned analytes in drinking water. The uncertainty of the measurement was 40%, which is lower than the requirement specified in the Spanish directive (RD 3/2023).

**Table 3:** Method calibration linear ranges, limits of detection, accuracy, and relative standard deviation at three quality control levels of 26 PFAS investigated in this study

Analyte	Linear range [ng/L]	^a^LOD [ng/L]	% accuracy Ebro River water			% accuracy drinking water		
			^b^5 [ng/L]	^b^50 [ng/L]	^b^100 [ng/L]	^b^5 [ng/L]	^b^50 [ng/L]	^b^100 [ng/L]
PFBA	2.5–100	0.8	128	110	115	118	104	119
PFBS	0.25–5 5–100	0.1	115	96	85	105	87	93
PFDA	0.25–5 5–100	0.1	78	80	105	88	88	95
PFDoA	0.25–5 5–100	0.1	120	93	104	110	85	94
PFDoS	2.5–100	0.8	76	84	128	71	94	122
PFDS	2.5–100	0.8	130	110	125	136	86	113
PFHpA	5–100	1.7	80	89	93	75	79	84
PFHpS	5–100	1.7	126	108	112	106	99	104
PFHxA	0.25–5 5–100	0.1	85	93	82	96	87	95
PFHxS	2.5–100	0.8	115	74	82	108	88	96
PFNA	0.25–5 5–100	0.1	88	78	75	94	82	88
PFNS	2.5–100	0.8	92	85	84	114	105	113
PFOA	0.25–5 5–100	0.1	111	115	118	99	83	89
PFOS	2.5–100	0.8	109	112	107	97	88	97
PFOSA	2.5–100	0.8	120	116	112	104	79	82
PFPeA	0.25–5 5–100	0.1	110	113	117	89	85	92
PFPS	2.5–100	0.8	130	125	123	130	113	107
PFTrA	2.5–100	0.8	81	73	70	92	81	78
PFTrS	5–100	1.7	-	72	81	-	83	97
PFUnDA	2.5–100	0.8	90	91	85	101	87	93
PFUnS	2.5–100	0.8	75	81	76	103	72	90
4:2 FTS	2.5–100	0.8	129	121	119	123	73	79
6:2 FTS	2.5–100	0.8	126	128	132	100	131	135
8:2 FTS	2.5–100	0.8	73	75	78	81	73	86
N-EtFOSAA	2.5–100	0.8	132	116	121	136	100	108
N-MetFOSAA	2.5–100	0.8	129	122	130	86	77	74

^a^LOD average between the studied waters

^b^%RSD (*n* = 5) < 20%

^*^LOQs were defined as the lowest point of each calibration curve with S/N ≥ 10, for each compound

The method accuracy met the expected criteria (70–130%) at three concentration levels (5, 50, and 100 ng/L) and as well as precision with RSD < 20% (*n* = 5). These results were comparable to those obtained with other recent methods (Coggan et al. 2019; Martin et al. 2019; Liu et al. 2020).

In the ERA proficiency test, 18 target analytes were determined in drinking water at concentrations ranging from < 60 to 486 ng/L (the intercomparison sample was diluted because the concentration exceeded 100 ng/L). The trueness of the obtained results was assessed using the *z*-score parameter for each analyte, achieving values from − 1.13 to 1.26, within the satisfactory interval of − 2 to 2, indicating the reliability of the presented method.

Overall, based on the validation parameters of the method, we can conclude that this method is suitable for the quantification of the PFAS under study in drinking and surface water within in the working ranges of 0.25–5 ng/L and 5–100 ng/L.

### Analysis of targeted PFAS

#### Occurrence in DWTP water samples

Twenty-six PFAS compounds were determined in water samples from different treatment processes of l’Ampolla DWTP per triplicate. Sampling locations were selected taking into account information from different studies in the bibliography that demonstrated that oxidation processes involving the use of ozone, GAC, and UV could decrease, enhance, or be ineffective in terms of PFAS concentration (Takagi et al. 2011; Rahman et al. 2014; Thomas et al. 2020; Verma et al. 2021a; Zhong et al. 2023).

**Fig. 2:**
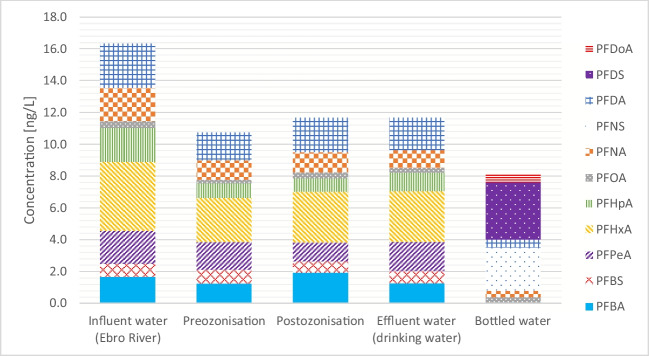
Total (∑20) PFAS average concentration in Ebro River water, pre-ozonation sample, post-ozonation sample, drinking water, and bottled water

Of 26 PFAS compounds monitored in the present study (20 of them included in RD 3/2023 to be regulated until January 2025), 17 were detected at least once in water samples from different water treatment stages at the l’Ampolla DWTP (Ebro River water, preozonation, postozonation, and drinking water), with detection frequencies of 25–100% (Table 4). As shown in Fig. 2, the ∑_20_ PFAS average concentration in samples from the DWTP ranged between 11 and 16 ng/L, which results are lower than 100 ng/L, the parametrical value specified in Spanish legislation (Ministerio de la Presidencia R con las C y MD 2023). Moreover, PFOA, PFNA, PFHxS, and PFOS concentrations in drinking water were 0.3 ng/L, 1.1 ng/L, and < LOQ for PFHxS and PFOS, respectively, all below the individual parametrical value of 70 ng/L for each compound) (Ministerio de la Presidencia R con las C y MD 2023). Regarding DWTP influent water, the obtained results (1.7 ng/L for PFBA, 0.8 ng/L for PFBS, 0.4 ng/L for PFOA, and < LOQ for PFOS) are in line with a study of the literature regarding PFAS content in samples from areas without industrial influence. In this case, the results obtained were < 5 ng/L for PFOA and < LOQ (0.6 ng/L) for PFBS, PFDA, PFNA, PFOS, and PFTeA (Castiglioni et al. 2015).

**Table 4:** Summary of PFAS occurrence (% frequency of detection) and concentration in Ebro River water, water samples from DWTP and drinking water

Compounds	Influent water (Ebro River)				Preozonisation				Postozonisation				Effluent water (drinking water)			
	Freq	Min [ng/L]	Max [ng/L]	Mean [ng/L]	Freq	Min [ng/L]	Max [ng/L]	Mean [ng/L]	Freq	Min [ng/L]	Max [ng/L]	Mean [ng/L]	Freq	Min [ng/L]	Max [ng/L]	Mean [ng/L]
PFBA	100	< MQL	3.1	1.7	100.0	< MQL	2.3	1.3	100.0	< MQL	3.6	1.9	100.0	< MQL	2.3	1.3
PFBS	100	0.7	1.0	0.8	100.0	0.7	1.0	0.9	100.0	0.4	0.9	0.7	100.0	0.3	1.0	0.7
PFPeA	100	1.5	2.6	2.1	100.0	1.4	2.2	1.7	100.0	0.0	2.0	1.2	100.0	1.2	2.5	1.8
PFPS	100	< MQL	< MQL		50.0	< MQL	< MQL		50.0	< MQL	< MQL		50.0	< MQL	< MQL	
PFHxA	100	3.2	5.5	4.3	100.0	1.6	3.2	2.8	100.0	2.9	3.6	3.2	100.0	1.7	4.2	3.2
PFHxS	100	< MQL	< MQL		25.0	< MQL	< MQL		100.0	< MQL	1.8	1.1	75.0	< MQL	< MQL	
PFHpA	100	0.2	3.4	2.1	100.0	0.1	1.8	0.9	100.0	0.2	1.9	0.8	100.0	0.0	2.5	1.2
PFHpS	100	< MQL	< MQL		100.0	< MQL	< MQL		100.0	< MQL	< MQL		100.0	< MQL	< MQL	
PFOA	100	< MQL	0.7	0.4	100.0	< MQL	0.3	0.2	100.0	< MQL	0.6	0.4	100.0	< MQL	0.5	0.3
PFOS	100	< MQL	< MQL		75.0	< MQL	< MQL		75.0	< MQL	< MQL		75.0	< MQL	< MQL	
PFNA	100	1.3	2.7	2.1	100.0	1.1	1.3	1.2	100.0	1.2	1.5	1.3	100.0	1.0	1.2	1.1
PFNS	100	< MQL	< MQL		100.0	< MQL	< MQL		100.0	< MQL	< MQL		100.0	< MQL	< MQL	
PFDA	100	1.3	3.6	2.8	100.0	< MQL	2.9	1.8	100.0	1.5	2.8	2.2	100.0	1.5	2.7	2.1
PFDS	50	n.d	< MQL		50.0	n.d	< MQL		50.0	n.d	< MQL		50.0	n.d	< MQL	
PFDoA	0	n.d	n.d		0	n.d	n.d		0	n.d	n.d		0	n.d	n.d	
PFDoS	100	< MQL	< MQL		100	< MQL	< MQL		100	< MQL	< MQL		100	< MQL	< MQL	
PFUnDA	0	n.d	n.d		0	n.d	n.d		0	n.d	n.d		0	n.d	n.d	
PFUnS	100	< MQL	< MQL		100	< MQL	< MQL		100	< MQL	< MQL		100	< MQL	< MQL	
PFTrA	0	n.d	n.d		0	n.d	n.d		0	n.d	n.d		0	n.d	n.d	
PFTrS	0	n.d	n.d		0	n.d	n.d		0	n.d	n.d		0	n.d	n.d	
4:2 FTS	0	n.d	n.d		0	n.d	n.d		0	n.d	n.d		0	n.d	n.d	
6:2 FTS	100	< MQL	< MQL		100	< MQL	< MQL		100	< MQL	< MQL		100	< MQL	< MQL	
8:2 FTS	0	n.d	n.d		0	n.d	n.d		0	n.d	n.d		0	n.d	n.d	
N-MetFOSAA	0	n.d	n.d		0	n.d	n.d		0	n.d	n.d		0	n.d	n.d	
N-EtFOSAA	0	n.d	n.d		0	n.d	n.d		0	n.d	n.d		0	n.d	n.d	
PFOSA	0	n.d	n.d		0	n.d	n.d		0	n.d	n.d		0	n.d	n.d

The behavior of PFAS in the DWTP processes was investigated. In general, PFAS concentration (taken as the average for the studied period) decreased by approximately 30% between the DWTP effluent water and influent water of the same plant. This reduction can be attributed to the different treatment processes that take place there. In this study, the DWTP ozone dosage varied between 0.4 and 0.6 g/m^3^, with water pH in pre- and post-ozonation stages maintained at 7.5. These conditions could potentially oxidize the present PFAS compounds, as similar operation conditions in the literature have shown an increase in PFAS concentration after pre-ozonation (0.5 g/m^3^) (Verma et al. 2021b). Nonetheless, as observed in Fig. 2, water samples collected after the pre- and post-ozonation steps show minimal variations in PFAS concentrations under typical DWTP conditions. However, the values for individual PFAS activities obtained are close the LOQ, which must be considered when assessing PFAS removal/oxidation at this stage. After post-ozonation, the treated water entered the GAC unit. In general, long-chain PFAS are removed in the GAC unit due to their high hydrophobicity, while shorter PFAS are less effectively removed (Rahman et al. 2014; Wang et al. 2020). In L’Ampolla DWTP, water enters a UV-disinfection process (*λ* = 254 nm) after the GAC unit. As observed in Table 4, both the influent and effluent of the DWTP have low or no concentrations of long-chain PFAS. However, short-chain PFAS were detected in the influent but were not removed by GAC. The PFAS compound concentrations found in the surface water used as DWTP raw water were at low ng/L levels and, at the specific time when the study was conducted, the activated carbon was aged (Rahman et al. 2014). For these reasons, it is not possible to appreciate the removal efficiency of the GAC followed by the UV-disinfection process (*λ* = 254 nm) conclusively in this study.

#### Occurrence in bottled water samples

As regards bottled waters, ten natural mineral bottled waters, contained in polyethylene terephthalate bottles, from different Spanish sources were analyzed. Only 7 PFAS compounds were positively detected, with concentrations below LOQ (PFBA, PFBS, PFHxA, PFHpA, PFHpS, and PFDoA) in 9 of the ten samples analyzed. However, as shown in Fig. 2, one sample showed the presence of 0.5 ng/L for PFOA, 0.4 ng/L for PFNA, 2.7 ng/L for PFNS, 0.5 ng/L for PFDA, 3.6 ng/L for PFDS, and 0.5 ng/L for PFDoA (RSD% < 15% for *n* = 3) total PFAS content being 8 ng/L. The quantified PFAS compounds have analogue ISTD, so no correction has been applied in terms of matrix effect. The concentration of PFAS in bottled water varies between studies from different countries around the world, from < LOQ to 15 ng/L, which are results in line with those presented in this study, and in general lower than those obtained for drinking water (Teymoorian et al. 2022). The differences in PFAS concentrations between drinking water from a DWTP and bottled water are probably due to water origin. In this case, drinking water is produced by treating river water, which can be influenced by industrial or wastewater treatment plant effluents, while bottled water typically comes from groundwater sources where PFAS contamination is limited.

### Analysis of PFAS precursors

One consideration that should be taken into account when performing a TOP assay is the evaluation of the oxidation efficiency, as explained in the “Method performance evaluation” section. In a first attempt, using the same doses of oxidant as used by Houtz and Sedlak (2012), the presence of 6:2 FTS was observed after TOP assay, which indicated an incomplete oxidation, and which implies an underestimation of precursor concentration. This fact may be related to the presence of co-contaminants, sample organic content, halide ions, carbonates, or humic acid (Ateia et al. 2023). As shown in Table 5, two different oxidant dosages conditions were tested. In both cases, no precursor was determined after the TOP assay, and the ratio between the sum of concentrations of PFAA precursors and the sum of total PFAS was less than 5%, which indicated a successful oxidation (Nolan et al. 2019). Moreover, the molar conversion yields of 6:2 FTS obtained were in line with those from the literature. One hundred eighty millimolar of persulfate and 450 mM of NaOH were the suitable dosage that fully oxidized 6:2 FTS in the surface water under study.

**Table 5:** Molar conversion yields of 6:2 FTS into C4-C7 perfluoro carboxylic acids through the TOP-SPE method in surface water. Oxidation dosage conditions were modified versus those from the literature

		This study (surface water)		(Martin et al. 2019)	(Houtz and Sedlak 2012)
Concentration [mM]	Persulfate	180	450	60	60
	NaOH	450	900	150	150
Molar yield [%]	PFBA	15 ± 3	15 ± 5	15 ± 4	22 ± 5
	PFHxA	19 ± 2	18 ± 1	15 ± 0.3	22 ± 2
	PFHpA	2 ± 1	3 ± 1	2 ± 0.3	2 ± 1
	PFPeA	16 ± 4	16 ± 3	35 ± 11	27 ± 2

After that, the presence of PFAS precursors was evaluated. In the presented analytical method, some precursors have been included as targeted compounds (4:2 FTS, 6:2 FTS, 8:2 FTS, PFOSA, N-MetFOSAA, and N-EtFOSAA) and only one of them, 6:2 FTS, was detected in L’Ampolla water samples at < LOQ concentration before the TOP assay. Specifically, this compound oxidizes to form multiple PFCAs, such as PFBA, PFHxA, PFHpA, and PFPeA (Houtz and Sedlak 2012; Martin et al. 2019). In summary, after performance of the TOP assay, 6:2 FTS was not detected, so a near-complete oxidation of this compound, and possibly of unknown precursors, has taken place. Because of this, some PFCAs suffered qualitatively detectable low increases in terms of molar concentration. However, and as has been stated in a previous study, samples with lower total precursor molar concentrations, as is the case of the present research, have significant associated uncertainties with PFCA molar concentrations (Pickard et al. 2022). Thus, the TOP assay performed, reinforced by the results of the previous targeted analysis, provides qualitative information on the presence of precursors in water samples at low concentrations, < 2.5 ng/L, such as 6:2 FTS.

Regarding bottled water, no target precursors included in the method have been detected. Moreover, the TOP assay did not reveal qualitative increases in terms of PFCAs concentration, confirming the absence of other precursors in these water samples.

### Exposure and human health risk assessment for PFAS

The estimated weekly intake for the sum of PFOA, PFNA, PFHxS, and PFOS has been determined for two age groups. As shown in Table 6, the exposure for adults, children, and infants suggests that drinking water consumption is responsible for no more than 10%, 21%, and 46% of TWI, respectively. For bottled water, the EWI are below the TWI threshold by a factor percentage of 7%, 21%, and 32% respectively. So, water ingestion of the analyzed samples should not pose any risk to the population. The presented results are in line with those reported by Jurikova et al. (2022), where the evaluation of the ingestion of Czech drinking water samples containing PFAS showed that the recommended guidelines were not exceeded.

**Table 6:** Estimated weekly intake calculated for adults, child, and infants due to the ingestion of drinking water and bottled water (values obtained in this study) for ∑ PFOA, PFNA, PFHxS, and PFOS

Sample type	Concentration [ng/L]	EWI_adult_ (ng/kg_bw_ per week]	EWI_children_ (ng/kg_bw_ per week]	EWI_infant_ (ng/kg_bw_ per week]
Drinking water	1.9	0.4	1.3	2.0
Bottled water	1.1	0.3	0.9	1.4

*EWI* estimated weekly intake

*TWI* tolerable weekly intake

**Fig. 3:**
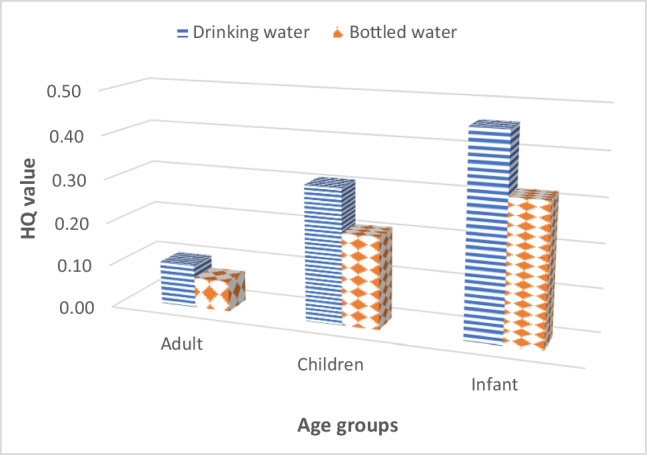
Calculated HQ values for average concentration of the sum of PFOA, PFNA, PFHxS, and PFOS. Calculations were performed for different age groups derived from ingestion drinking water from l’Ampolla DWTP and Spanish bottled water

The potential health risks associated with drinking water ingestion containing PFOA, PFNA, PFHxS, and PFOS has been evaluated using hazardous quotients. As shown in Fig. 3, overall, considering DWTP water consumption, HQ values fell within the range of 0.1–1, indicating a low hazard for all age groups. For bottled water, the HQ for adults was < 0.1, representing no hazard. However, for children and infants, the HQ indicated low hazard. It can be concluded that the population exposed to PFAS through ingestion of the mentioned waters does not exceed the current EFSA intake recommendation, suggesting that it may not represent a potential health risk.

Finally, a worst-case scenario considering the individual parametrical values for PFOA, PFNA, PFHxS, and PFOS stated in the Spanish Royal Decree 3/2023, set at 70 ng/L for each compound, is contemplated to assess if these stablished values might cause adverse effects on human health for each group studied or are restrictive enough. As observed in Table 7, drinking water containing a total concentration of 280 ng/L considerably exceeds the TWI value set by EFSA in each age group, and the related HQs were > 10, indicating that the ingestion of water containing this PFAS concentration may pose a high hazard to human health. This is because the parametrical values stablished in the Spanish or European drinking water directive have no direct connection to EFSAs TWI value for the mentioned fourth PFAS, unlike PFASs levels in food. In this case, it is recommended to revise and stablish new limit values for these compounds, as done in Denmark with a value of 2 ng/L for the sum of these four PFAS (which is derived from the EFSA TWI safe drinking water threshold) in order to ensure sufficient protection for vulnerable groups, avoiding negative effects (Johansson and Hermann 2023).

**Table 7:** EWI and HQ calculated for the individual parametric value of PFOA, PFNA, PFHxS, and PFOS stablished in the Spanish RD 03/2023

Concentration [ng/L]	EWI_adult_ (ng/kg_bw_ per week]	EWI_children_ (ng/kg_bw_ per week]	EWI_infant_ (ng/kg_bw_ per week]	HQ_adult_	HQ_children_	HQ_infant_
280.0	65.3	196.0	294.0	14.8	44.5	66.8

In view of the obtained results, it is advisable to control the presence of these substances in drinking water, but attention should also be paid to reducing the exposure of the population to PFAS, especially through diet and other routes of exposure, like indoor air inhalation, dust ingestion, and dermal absorption (Domingo and Nadal 2019; Poothong et al. 2020; DeLuca et al. 2022; Ragnarsdóttir et al. 2022).

## Conclusions

In this study, an analytical method based on off-line SPE and HPLC–MS/MS has been implemented for the determination of 26 targeted PFAS in Ebro River water, water from pre- and post-ozonation from l’Ampolla DWTP, drinking water and bottled water. Moreover, the TOP assay has been used to assess PFAS precursors. The current methodology employs isotopically labelled compounds as surrogates to compensate SPE recoveries and matrix effect. Although it was impossible to achieve the same labelled compound for each targeted PFAS, the analogues chosen performed, in general, good quantifications. LOQs are in the range of 0.25–5 ng/L, which are optimal for providing information on PFAS content in Spanish drinking water as they are in line with the current Spanish regulation (RD 3/2023). The method was validated and is capable of determining 26 PFAS compounds in different water samples. Firstly, samples from l’Ampolla DWTP were analyzed, presenting low PFAS concentrations (12–16 ng/L). Only 8 targeted compounds were quantified > LOQ. Due to the extremely low concentration of found PFAS, this makes them unlikely compounds to observe a reduction through the treatment processes of the DWTP. As regards bottled water, only one of ten commercial brands analyzed present PFAS (ng/L). The presence of 6:2 FTS (< LOQ) precursors in the mentioned samples has been determined by performing a TOP assay and reveals the absence of any other PFAS precursor in the analyzed sample. Finally, the results obtained were used for estimating health exposure and human health risk, concluding that there is no risk from consuming DWTP drinking water or bottled water.

## Data Availability

All data supporting the findings of this study is available from the corresponding author on reasonable request.
